# Expanded Perlite Reinforced Magnesium Phosphate Cement-Based Fireproof Coating: Composition Optimization, Fire Resistance and High-Temperature Phase Evolution Mechanism

**DOI:** 10.3390/ma19081492

**Published:** 2026-04-08

**Authors:** Runqing Liu, Chunyu Wang, Yuxin Ling

**Affiliations:** 1 School of Architecture and Civil Engineering, Liuzhou Institute of Technology, Liuzhou 545616, China; 2 Liaoning Ruifeng New Building Materials Co., Ltd., Tieling 112000, China; 3 School of Materials Science and Engineering, Shenyang Ligong University, Shenyang 110159, China

**Keywords:** inorganic fireproof coating, lightweight aggregate, thermal insulation, ceramic skeleton, phase transformation, bond strength, MPC composite

## Abstract

To develop a high-performance inorganic fireproof coating suitable for steel structures, this study utilized magnesium phosphate cement (MPC) as the matrix and introduced expanded perlite (EP) as a lightweight aggregate. The effects of EP content (40–55%) and magnesium-to-phosphorus ratio (M/P = 4:1–7:1) on the dry density, compressive strength, bond strength, and fire resistance of the coating were systematically investigated. X-ray diffraction (XRD), scanning electron microscopy (SEM), and thermogravimetric analysis (TGA) were employed to reveal the phase evolution and microstructure evolution mechanisms at high temperatures. The results indicate that increasing EP content significantly reduces the dry density and thermal conductivity of the coating, enhancing thermal insulation performance. However, excessive incorporation leads to the deterioration of mechanical properties, with an optimal EP content of 45%. The M/P ratio influences the interfacial bond strength and high-temperature structural stability by regulating the proportion of the hydration product K-struvite (KMgPO_4_·6H_2_O) and residual MgO. Compressive strength peaked at M/P = 6:1 (0.80 MPa), while bond strength was optimal at M/P = 5:1 (0.097 MPa), corresponding to the best fire resistance (back-side temperature of 180.4 °C). At high temperatures, K-struvite dehydrates and transforms into anhydrous KMgPO_4_, which, together with residual MgO and crystallized SiO_2_ from EP, forms a dense ceramic skeleton, ensuring the structural integrity of the coating. Comprehensive performance evaluation determined the optimal mix ratio as M/P = 5:1 and EP content = 45%. The coating with this ratio exhibits a dry density of approximately 560 kg/m^3^, a 14-day compressive strength of 0.53 MPa, a bond strength of 0.097 MPa, and a back-side temperature of 180.4 °C under flame exposure, demonstrating a favorable balance of lightweight character, mechanical integrity, and thermal insulation performance suitable for steel structure fire protection applications.

## 1. Introduction

Steel structures are widely used in modern buildings due to their light weight, high strength, and excellent seismic performance. However, the mechanical properties of steel degrade significantly at elevated temperatures, with yield strength and the elastic modulus beginning to decline above 300 °C and load-bearing capacity substantially lost at around 600 °C [1]. Therefore, effective fire protection is critical for steel structures. Among various fire protection measures, fireproof coatings are the most widely adopted due to their convenient construction and cost-effectiveness. Current fireproof coatings fall into two main categories: organic intumescent coatings and inorganic thick coatings. Organic intumescent coatings form an insulating carbonaceous layer upon heating but are prone to releasing toxic fumes and exhibit poor durability after aging [2]. Traditional inorganic thick coatings, such as cement-based materials, are non-toxic and environmentally friendly but suffer from weak bonding to steel substrates and susceptibility to cracking under thermal stress [3]. Consequently, developing inorganic fireproof coatings that combine strong bonding, high-temperature stability, and excellent thermal insulation is of significant theoretical and practical importance.

Magnesium phosphate cement (MPC) is a promising binder for such applications. MPC hardens rapidly through an acid–base reaction, with K-struvite (KMgPO_4_·6H_2_O) as its primary hydration product [4]. It offers high early strength, excellent bonding to steel, good volume stability, and corrosion resistance [5,6]. Under high-temperature exposure, MPC undergoes phase transformation: K-struvite dehydrates to form anhydrous KMgPO_4_, which together with residual MgO creates a ceramized skeleton that maintains structural integrity [7,8]. However, pure MPC paste has relatively high thermal conductivity (0.5–1.0 W/(m·K)), limiting its insulation efficiency [9]. To address this, lightweight porous aggregates such as expanded perlite (EP) are introduced. EP is characterized by low bulk density (50–200 kg/m^3^), extremely low thermal conductivity (0.04–0.06 W/(m·K)), and high-temperature resistance (softening point: 900–1100 °C) [10,11], making it an ideal aggregate for thermal insulation materials [12,13]. Preliminary studies have shown that incorporating EP into MPC can effectively reduce density and improve thermal insulation [14].

Despite these advances, several critical research gaps remain. First, the optimal EP content range in MPC-based fireproof coatings has not been systematically established; the comprehensive effects of EP content on workability, mechanical strength, and fire resistance require further investigation. Second, the magnesium-to-phosphorus (M/P) ratio determines the proportion of K-struvite and residual MgO, directly affecting matrix density, mechanical properties, and high-temperature stability [15,16]. However, the synergistic regulation mechanism between EP content and M/P ratio in the composite system remains underexplored. Third, while the high-temperature phase evolution of pure MPC has been studied [7,8], the behavior of EP-MPC composites—particularly regarding the crystallization of amorphous SiO_2_ from EP and its contribution to the ceramic skeleton—requires in-depth investigation [17]. Recent studies have advanced the understanding of MPC-based fireproof coatings, including the use of waste magnesia–carbon bricks [18], brucite-based MPC [19], the effect of phosphate-to-magnesium ratio [20], the incorporation of sulphoaluminate cement for enhanced high-temperature performance [21], and glass fiber reinforcement [22]. Additionally, the modification of EP in MPC mortars has been explored to improve interfacial bonding [14]. However, the synergistic effects of EP content and M/P ratio on coating performance have not been systematically addressed, and the structure–activity relationship linking phase evolution, microstructure, and fire resistance remains to be elucidated [23].

To fill these gaps, this study systematically investigates the synergistic effects of EP content (40–55%) and M/P ratio (4:1–7:1) on the dry density, compressive strength, bond strength, and fire resistance of MPC-based fireproof coatings. The objectives are to: (1) determine the optimal EP content that balances thermal insulation and mechanical performance; (2) reveal the influence mechanism of M/P ratio on interfacial bond strength and high-temperature structural stability; and (3) establish the structure–activity relationship between phase evolution, microstructure, and fire resistance under high-temperature exposure. Macro-performance tests are combined with microstructural characterization (XRD, SEM, TG) to elucidate the underlying mechanisms. This study provides a theoretical basis and experimental guidance for the composition optimization of EP-reinforced MPC fireproof coatings for steel structures.

## 2. Raw Materials and Experimental Methods

### 2.1. Raw Materials

(1) Dead Burnt Magnesia

Dead burnt magnesia (MgO) is produced by calcining magnesite at a high temperature of 1700 °C and appears brownish-yellow. The dead burnt magnesia raw material used in this experiment was sourced from Haicheng, Liaoning Province, China. The particle size distribution of the dead burnt magnesia was determined by laser diffraction, with a D_50_ of 32.5 μm and a specific surface area of 0.42 m^2^/g. The MgO content was 97.1%, with the balance consisting primarily of SiO_2_, CaO, and Fe_2_O_3,_ as determined by X-ray fluorescence (XRF) analysis. The chemical composition is shown in Table 1.

(2) Potassium Dihydrogen Phosphate

Potassium dihydrogen phosphate (KH_2_PO_4_, abbreviated as KDP), analytically pure, was produced by Tianjin Damao Chemical Reagent Factory, with a stated purity of not less than 99.5% as per the manufacturer’s specification. The chemical composition of potassium dihydrogen phosphate is shown in Table 2.

(3) Borax

Borax was used as a retarder to slow down the reaction rate between dead burnt magnesia and phosphate, with a purity of 99.5%, produced by Tianjin Damao Chemical Reagent Factory (Tianjin, China). The borax was added at a fixed dosage of 10% by weight of MgO for all mix proportions, based on preliminary optimization experiments.

(4) Mixing Water

The water used in this experiment was tap water.

(5) Expanded Perlite

Expanded perlite was produced by Xinyang Caster Technology Co., Ltd. (Xinyang, China), a lightweight, porous inorganic non-metallic material formed by the instantaneous high-temperature expansion of natural perlite ore. The expanded perlite particles had an irregular shape with a particle size range of 0.5–5.0 mm, with 70–90% of particles falling within the 1–3 mm range. The bulk density was measured as 60 kg/m^3^, and the water absorption rate (24 h immersion) was determined to be 280% by weight. Its parameters and chemical composition are shown in Table 3 and Table 4.

### 2.2. Mix Proportion Design

This study investigated the influence of the aggregate-to-matrix ratio and the magnesium-to-phosphorus ratio on coating performance, using the mass ratio of expanded perlite to magnesium phosphate cement as the research variable. First, expanded perlite was used as the main lightweight aggregate, with its mass fractions set at 40%, 45%, 50%, and 55%, corresponding to magnesium phosphate cement matrix mass fractions of 60%, 55%, 50%, and 45%, respectively. The magnesium-to-phosphorus ratio was kept constant during this process to analyze the influence of the aggregate-to-matrix ratio on coating performance. Second, with the selected expanded perlite content of 45%, the magnesium-to-phosphorus ratio was adjusted to 4:1, 5:1, 6:1, and 7:1 to investigate the influence of M/P ratio variation on coating workability, mechanical properties, and high-temperature stability. The specific water demand refers to Section 3.1 and Section 3.2 (calculated based on fluidity). For all mix proportions, borax was added at a fixed dosage of 10% by weight of MgO (i.e., 10% of the dead burnt magnesia mass), based on preliminary optimization experiments. The borax content is not included in the mass percentages listed in Table 5, as it is calculated separately based on the MgO content. The specific mix proportions are shown in Table 5.

### 2.3. Specimen Preparation

Three main specimen sizes were prepared in this experiment: (1) 70.7 mm × 70.7 mm × 70.7 mm, primarily used for testing dry density and compressive strength; (2) 40 mm × 40 mm × 10 mm, primarily used for testing bond strength; (3) 150 mm × 70 mm × 10 mm, primarily used for testing fire resistance. For bond strength and fire resistance tests, the Q235 steel plate substrates (Jiangsu Shagang Group Co., Ltd., Zhangjiagang, China) were prepared by sandblasting to remove surface oxides and achieve a uniform surface roughness, followed by cleaning with acetone to eliminate residual oil and contaminants.

According to the mix proportions in Table 5, predetermined amounts of expanded perlite, dead burnt magnesia, potassium dihydrogen phosphate, and borax were weighed and dry-mixed. A planetary mortar mixer (Model JJ-5, manufactured by Wuxi Jianyi Instrument Co., Ltd., Wuxi, China) was used for all mixing procedures. The dry materials were mixed at low speed (140 ± 5 rpm) for 60 s to ensure uniform distribution of components. The amount of mixing water was determined based on achieving a target fluidity of 260 ± 5 mm, as described in Section 3.1. For each mix proportion, the water consumption was pre-determined from the water demand tests and is summarized in Table 6. Then, mixing water was added, the mixture was mixed at low speed (140 ± 5 rpm) for 30 s, followed by mixing at high speed (285 ± 10 rpm) for 90 s to obtain the magnesium phosphate cement mortar. After high-speed mixing, the mortar was mixed at low speed for an additional 2–3 min to eliminate large air bubbles and ensure homogeneity. The total mixing time was controlled between 15 min and 20 min to account for the retarding effect of borax and to maintain consistent workability across all batches. The uniformly mixed coating was placed into molds. The molds were pre-coated with a thin layer of oil on the bottom and inner walls to facilitate demolding. Tamping was used to eliminate internal air bubbles. Excess coating above the mold surface was scraped off and leveled with a plastering trowel. Specimen preparation and curing were carried out under ambient conditions of temperature of 5 °C to 35 °C and relative humidity of 50% to 80%. To ensure consistency across all batches, the ambient temperature was controlled at 23 ± 2 °C and relative humidity at 60 ± 5% during specimen preparation. Curing conditions were continuously monitored using a temperature–humidity data logger (Model TH-10, manufactured by Elitech, San Jose, CA, USA) throughout the curing period. All specimens were cured in the same environmental chamber to minimize variability due to environmental fluctuations. After 24 h of curing, the specimens were demolded and continued curing until the specified age for relevant testing.

### 2.4. Experimental Methods

#### 2.4.1. Fluidity

According to GB/T 2419-2005 [24], “Test method for fluidity of cement mortar,” this experiment used a cement mortar fluidity table to measure fluidity for determining the water requirement of the MPC-based steel structure fireproof coating. The fluidity test was conducted using a jumping table apparatus (Model NLD-3, manufactured by Wuxi Jianyi Instrument Co., Ltd.). Each mix proportion was tested three times, and the average value was reported as the final fluidity. The target fluidity range for water demand determination was 260 ± 5 mm, consistent with the requirements for spray-applied fireproof coatings.

#### 2.4.2. Dry Density

The materials mixed according to the mix proportion were placed into 70.7 mm × 70.7 mm × 70.7 mm molds, gently shaken, tamped, and leveled with a tool. After basic drying and solidification, they were demolded. They were cured in a designated curing room (ambient temperature of 5–35 °C, relative humidity of 50–80%) for 3 d, 7 d, and 14 d. After the curing period, the specimens were placed in an electrothermal blowing dry box at 60 ± 5 °C for 48 h and then removed and cooled to room temperature. Using a caliper and electronic balance, the volume and mass of each specimen were measured. The dry density was calculated as the mass divided by the volume, with each value representing the average of three parallel specimens (n = 3). The standard deviation was calculated to assess the variability of the measurements.

#### 2.4.3. Compressive Strength

The compressive strength test was conducted according to Chinese National Standard GB/T 17671-2021 [25], “Test method of cement mortar strength (ISO method)”. The specimens were removed from the curing room at the specified age (3, 7, and 14 days), and their surfaces were cleaned. A microcomputer-controlled electronic universal testing machine (Model WDW-100, manufactured by MTS Systems Corporation (Eden Prairie, MN, USA), with a maximum load capacity of 100 kN) was used for loading. The test was performed under displacement control mode, with a constant loading rate of 0.5 mm/min, following the standard requirement for mortar strength testing. Three cubic specimens (70.7 mm × 70.7 mm × 70.7 mm) were tested for each mix proportion at each curing age. The compressive strength was calculated as the maximum load divided by the cross-sectional area (5000 mm^2^). The reported values represent the average of three replicate specimens, and error bars in the figures represent one standard deviation. Outliers were identified and excluded using Grubbs’ test with a significance level of α = 0.05. After outlier removal, the mean and standard deviation were recalculated based on the remaining valid measurements.

#### 2.4.4. Bond Strength

The bond strength test was conducted according to Chinese National Standard GB/T 5210-2006 [26], “Determination of tensile adhesion strength of coatings”. The substrate used was a Q235 steel plate of size 70 mm × 70 mm × 6 mm. Prior to coating application, the steel plate surface was cleaned with acetone to remove oil and contaminants, followed by sandblasting to achieve a surface roughness of approximately Ra 5–10 μm, which enhanced mechanical interlocking between the coating and the steel substrate. The molding frame specifications were 40 mm × 40 mm × 10 mm, corresponding to a coating thickness of 10 mm after application. The mixed coating was evenly applied inside the molding frame. After specimen formation, the molding frame was removed. Curing was carried out under controlled conditions of temperature of 23 ± 2 °C and relative humidity of 60 ± 5% to ensure consistency across all specimens. After the curing period, a two-component epoxy adhesive (E-44 epoxy resin with polyamide curing agent) was evenly brushed on top of the coating layer. A steel connector with a 40 mm × 40 mm loading surface was then adhered, and a 1 kg weight was pressed on top to ensure uniform contact. Excess epoxy resin around the edges was wiped off. The adhesive was allowed to cure for 24 h before the bond strength test. Tensile force was applied vertically along the baseplate direction at a constant loading rate of 1500 N/min using the same universal testing machine (Model WDW-100) (Shanghai Precision Instrument Co., Ltd., Shanghai, China). The bond strength of the specimen was calculated according to formula (3). To ensure that failure occurred at the coating–substrate interface rather than within the adhesive layer, preliminary tests were conducted to verify that the epoxy adhesive strength exceeded the bond strength of the coating; in all cases, failure occurred at the coating–steel interface. The bond strength result is expressed as the average value of 5 test values after eliminating outliers using Grubbs’ test (also known as the extreme studentized deviate test) with a significance level of α = 0.05. This objective statistical method identifies outliers as values that deviate significantly from the mean; any data point exceeding the critical Grubbs’ statistic was excluded from the final calculation.

*f_b_* = F/A (1)

where: *f_b_*—bond strength of the specimen, in MPa; F—maximum tensile load, in N; A—bonded area of the specimen, in mm^2^.

#### 2.4.5. Fire Resistance

**Figure 1:**
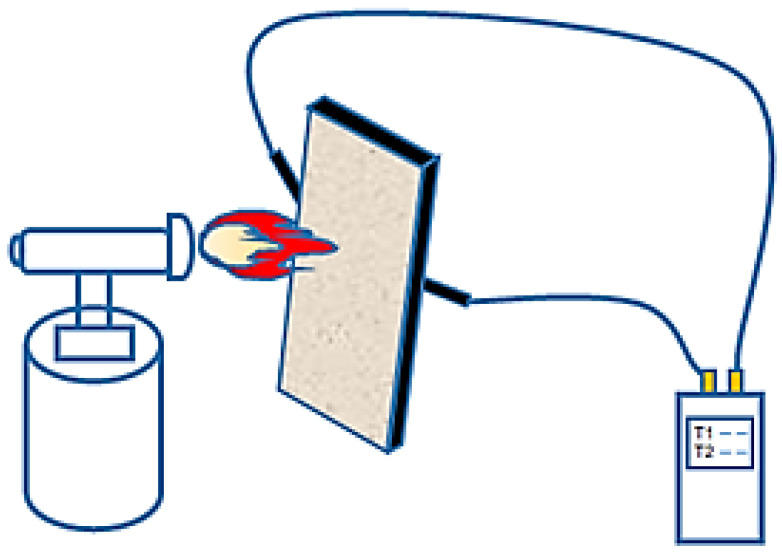
Schematic diagram of the fire resistance test setup.

Materials were weighed according to the designed mix proportion. The mixed coating was evenly applied onto a Q235 steel plate (Jiangsu Shagang Group Co., Ltd., Zhangjiagang, China) of the size 150 mm × 70 mm × 1 mm, with a controlled coating thickness of approximately 10 mm. After the coating dried and hardened, it was demolded, and the specimen was placed under ambient conditions of temperature of 5–35 °C and relative humidity of 50–80% for curing until the specified time. After curing, the specimen was securely fixed on the experimental platform. Two thermocouples were attached to the center position on the back-side of the steel plate using high-temperature resistant tape. The other end of the thermocouples was connected to a multi-channel temperature recorder for accurate acquisition and monitoring of temperature changes, as shown in Figure 1. The flame device was ignited to heat the central area of the coating, with a flame nozzle positioned 50 mm from the coating surface, producing a heating zone of approximately 50 mm in diameter. The fuel used in this experiment was butane, and the flame temperature was monitored using a K-type thermocouple placed at the flame-exposed surface, with a maximum temperature of 1200–1300 °C.

#### 2.4.6. Microstructure Performance Testing

(1) X-ray Diffraction Test

For XRD testing of magnesium phosphate cement-based coatings, samples were first stopped from hydration, crushed, dried, and then ground finely in an agate mortar to pass through a 200-mesh sieve. For samples after fire exposure, the coating was carefully detached from the steel plate. To investigate the phase evolution across the coating thickness, samples were collected from two distinct regions of the central heated zone (the area directly under the flame, approximately 50 mm × 50 mm, as shown in Figure 1): (1) the flame-exposed surface layer (the side directly facing the flame), and (2) the steel-contact interface layer (the side adhered to the steel plate). The collected samples were then ground into powder for XRD analysis. Samples before and after combustion were tested using an Ultima IV type X-ray diffractometer (XRD) (Rigaku Corporation, Tokyo, Japan) with a scanning angle range of 5° to 85° and a step size of 0.02°.

(2) Scanning Electron Microscope Test

For SEM observation, specimens were first sectioned to obtain representative samples from different regions. For samples after fire exposure, specimens were carefully cut to separate the flame-exposed end (the surface directly facing the flame) from the steel plate contact end (the interface adjacent to the steel substrate). These sections were then fractured to expose fresh surfaces for observation. The samples were immersed in anhydrous ethanol for 24 h to stop hydration, followed by drying in an electrothermal blowing dry box at 45 °C for 24 h.

Prior to observation, the sample surfaces were coated with a thin layer of gold using a sputter coater (Model E-1010, Hitachi, Chiyoda-ku, Tokyo) to enhance electrical conductivity and prevent charging effects during imaging. An S-3400N microcomputer-controlled scanning electron microscope (SEM, Hitachi High-Tech Corporation, Tokyo, Japan) was used to observe the microstructure of hydration products of the magnesium phosphate cement-based coating before and after combustion. The SEM was operated at an accelerating voltage of 15 kV, with working distances ranging from 10 to 15 mm. Observations were performed at various magnifications, typically ranging from 200× to 5000×, to capture both the overall distribution of hydration products and the detailed morphology of the microstructure.

(3) Simultaneous Thermal Analysis Test

To terminate hydration and preserve the phase composition at the designated curing age, representative sample blocks were taken from specimens both before and after combustion and immediately immersed in anhydrous ethanol for 24 h. This treatment removes free water and stops further hydration reactions. The sample blocks were then removed from the ethanol and placed in an electrothermal blowing dry box at 45 °C for 24 h to remove residual solvent. After drying, they were ground into powder using an agate mortar, with the powder fineness controlled to 200 mesh. An appropriate amount of sample was taken, and thermogravimetric analysis was performed using a TGA/DSC Netzsch STA-449F3 instrument (NETZSCH-Gerätebau GmbH, Selb, Germany), heating at a rate of 10 °C/min up to 1000 °C under a protective N_2_ atmosphere.

### 2.5. Data Processing and Uncertainty Analysis

To ensure the reliability and reproducibility of the experimental results, all tests were conducted with three parallel specimens for each mix proportion and each curing age. For bond strength, five parallel specimens were tested to account for higher variability inherent in adhesion tests. The mean value and standard deviation were calculated for each set of measurements. To ensure the statistical validity of the results, outliers were identified and removed using Grubbs’ test (extreme studentized deviate test) with a significance level of α = 0.05. This objective method calculates Grubbs’ statistic, G = |xᵢ − $\overset{-}{x}$|/s, for each data point; a value is considered an outlier if G exceeds the critical value for the given sample size at the chosen significance level. Measurement uncertainties were estimated based on instrument precision: ±5 kg/m^3^ for dry density, ±0.02 MPa for compressive strength, ±0.005 MPa for bond strength, and ±2 °C for fire resistance back-side temperature. All reported values in the figures and tables represent the mean ± standard deviation after outlier removal. For fire resistance specimens, coating thickness was measured at five points (four corners and center) using a digital thickness gauge (Model TT210, manufactured by Time Group Inc., Beijing, China) prior to testing. Specimens with thickness variation exceeding ±0.5 mm were discarded to ensure uniformity across all tests.

## 3. Results and Discussion

### 3.1. Water Demand

The content of expanded perlite (EP) significantly influences the water demand of magnesium phosphate cement (MPC)-based fireproof coatings. In this study, the water consumption of the paste at different EP contents was measured based on a fixed fluidity (260 ± 5 mm), and the results are shown in Table 6. As the EP content increased from 40% to 55%, the water consumption of the paste continuously rose from 285 kg/m^3^ to 392 kg/m^3^, an increase of 37.5%. This trend indicates that the introduction of EP significantly alters the rheological behavior of the MPC paste.

The effect of EP on water demand mainly stems from its unique microstructural characteristics. As a typical porous amorphous material, EP particles are filled with numerous closed and open micropores, resulting in a large specific surface area. In the initial mixing stage, these micropores rapidly absorb free water through capillary force, leading to an instantaneous decrease in the effective free water content within the paste, which is consistent with previous observations on the water absorption behavior of lightweight aggregates in cementitious systems [10,13]. It is noteworthy that the water absorption capacity of EP is sensitive to ambient humidity; under higher-relative-humidity conditions, EP particles may pre-absorb moisture from the environment, which can reduce the initial water demand during mixing. Conversely, under dry conditions, the water demand increases due to the greater suction capacity of the dry porous aggregate. In this study, all mixing and curing procedures were conducted under controlled conditions (23 ± 2 °C, 60 ± 5% RH) to minimize such variability. Simultaneously, the rough surface and irregular shape of EP particles increase the mechanical interlocking between particles, raising the yield stress and plastic viscosity of the paste. These two factors together cause the water required to achieve the same fluidity to increase significantly with higher EP content. It is worth noting that the water absorbed by EP is not entirely “lost”; some may be slowly released during the later curing stage, participating in the continuous hydration reaction of MPC, acting as “internal curing.” This effect helps improve the density of the interfacial transition zone but also implies that excessively high initial water consumption due to excessive EP content may lead to increased coating porosity, subsequently affecting mechanical properties (see Section 3.3).

From the perspective of mix proportion optimization, an EP content of 45% represents a critical turning point: the water consumption increases by 9.5% compared to 40%, while the increase accelerates significantly after the content exceeds 50% (50% is an increase of 11.5% compared to 45%, and 55% is an increase of 12.6% compared to 50%). This suggests that 45% might be the critical content balancing workability and hardened properties, providing an important basis for subsequent research.

### 3.2. Fluidity

The magnesium-to-phosphorus ratio (M/P ratio) is a key parameter determining the rheological properties of the magnesium phosphate cement paste. Under the conditions of fixed expanded perlite content (45%) and water consumption, the fluidity test results for pastes with different M/P ratios are shown in Table 7. As the M/P ratio increased from 4:1 to 7:1, the fluidity of the paste showed a continuous decreasing trend, gradually dropping from 270 mm to 251 mm, a reduction of approximately 7%.

This pattern arises from the regulatory effect of the M/P ratio on the hydration reaction progress and the microstructure of the MPC paste. When the M/P ratio is low (4:1), potassium dihydrogen phosphate is relatively abundant in the system. Magnesia particles partially dissolve and react with phosphate to form K-struvite crystals. There are fewer unreacted solid particles, and the internal frictional resistance within the paste is relatively low, thus exhibiting higher fluidity. This trend is consistent with previous studies on the rheological behavior of MPC systems, which reported that increasing the M/P ratio leads to higher viscosity and reduced fluidity due to the increased content of unreacted MgO particles [15,16]. As the M/P ratio increases, the magnesia content gradually rises, leading to more unreacted magnesia particles appearing in the system. These particles have rough surfaces, increasing the internal frictional resistance of the paste. Simultaneously, magnesia continuously dissolves in the acidic environment and reacts with phosphate. The continuously precipitating K-struvite crystals form a flocculated structure, further increasing the paste’s viscosity.

It is worth noting that when the M/P ratio increased to 7:1, the magnesia excess was most pronounced. Free water in the paste was absorbed by the surfaces of numerous unreacted particles, thickening the mix and maximizing the frictional resistance between particles. Simultaneously, the flocculation effect of hydration products was also most significant, leading to the lowest fluidity (251 mm). This phenomenon indicates that under high-M/P-ratio conditions, the paste tends towards early densification, and the setting time may shorten, directly impacting construction performance.

From a construction perspective, when the M/P ratio is between 5:1 and 6:1, the paste fluidity remains around 260 mm, ensuring a certain operation time while preventing layering or segregation caused by being too thin. This range provides a suitable workability foundation for the subsequent optimization of mechanical properties and fire resistance.

### 3.3. Dry Density

**Figure 2:**
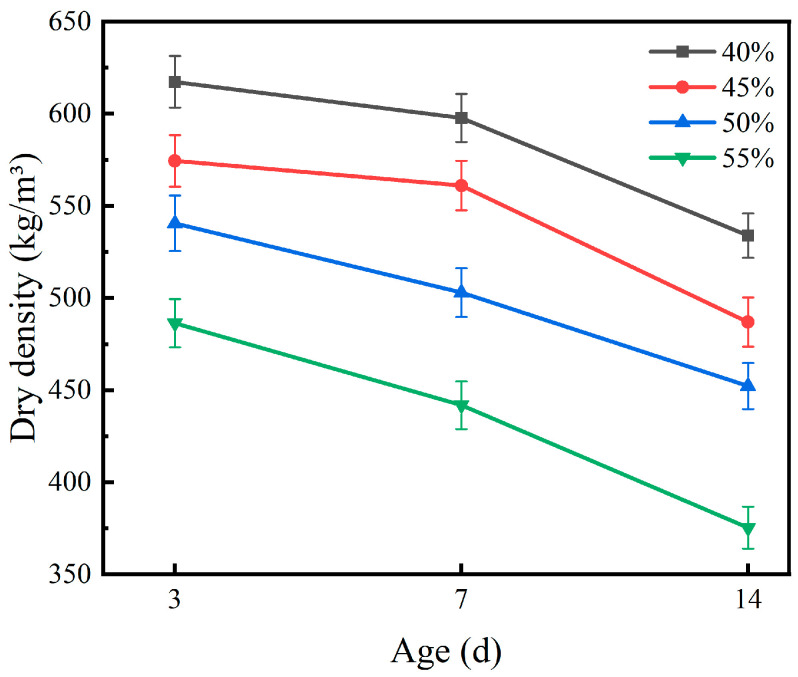
Effect of expanded perlite (EP) content on the dry density of MPC-based fireproof coatings at curing ages of 3, 7, and 14 days.

The effect of expanded perlite content on the dry density of the coating is shown in Figure 2. As the EP content increased from 40% to 55%, the dry density of the specimens showed a continuous decreasing trend. Taking the 3 d age as an example, the dry density at 40% EP content was 617.2 kg/m^3^, while at 55% content, it dropped to 486.3 kg/m^3^, a decrease of 21.2%. This pattern was also significant at 7 d and 14 d ages, indicating that the introduction of EP, as a lightweight porous aggregate, effectively reduces the overall compactness of the coating.

The mechanism by which EP reduces dry density stems from its porous structure. EP particles contain numerous closed and open micropores, with a bulk density of only 50–200 kg/m^3^, far lower than that of the MPC matrix. When EP content increases, the proportion of low-density components in the system increases, and the packing voids between particles also increase, leading to a rise in the overall porosity of the coating and a decrease in dry density. Similar trends have been reported in lightweight cementitious composites incorporating expanded perlite, where the dry density decreases proportionally with increasing EP content [8,11]. It is worth noting that at the same EP content, dry density further decreases with extended curing age. Taking the 40% content as an example, dry density decreased from 617.2 kg/m^3^ at 3 d to 533.8 kg/m^3^ at 14 d; for 55% content, it decreased from 486.3 kg/m^3^ to 375.2 kg/m^3^. This phenomenon is related to the evolution of moisture during curing: as the hydration reaction progresses, capillary water is gradually consumed and evaporates into the atmosphere. Some water is also adsorbed and retained by EP pores, leading to the continuous development of the material’s internal pore structure, and the measured dry density correspondingly decreases. The rate of moisture loss and the extent of dry density reduction are influenced by ambient conditions; higher temperatures accelerate moisture evaporation, while lower humidity promotes greater drying shrinkage. In this study, the controlled curing environment (23 ± 2 °C, 60 ± 5% RH) ensured consistent moisture evolution across all specimens, allowing for reliable comparison of dry density values under standardized conditions.

**Figure 3:**
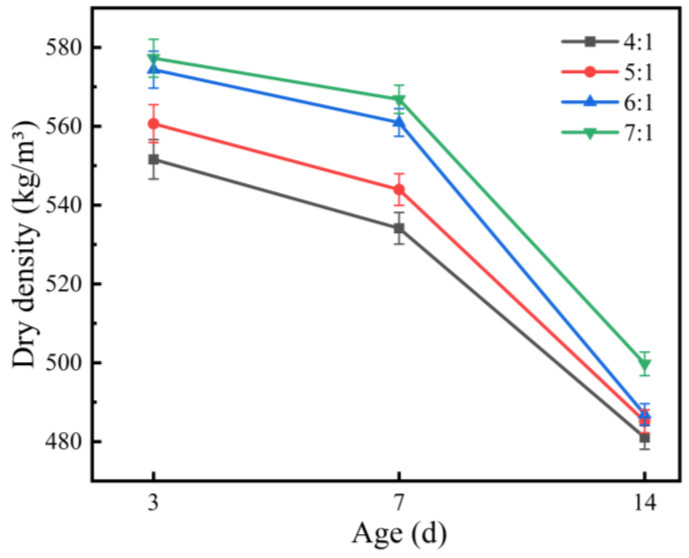
Effect of magnesium-to-phosphorus (M/P) ratio on the dry density of MPC-based fireproof coatings at curing ages of 3, 7, and 14 days.

The effect of the magnesium-to-phosphorus ratio on the dry density of the coating is shown in Figure 3. At the same curing age, dry density generally increased with an increasing M/P ratio. At 3 d, the dry densities for M/P ratios of 4:1, 5:1, 6:1, and 7:1 were 551.6, 560.6, 574.3, and 577.3 kg/m^3^, respectively, with significantly higher dry density under high-M/P-ratio conditions. This indicates that in high-magnesium systems, the reaction degree between magnesia and phosphate is higher, the amount of reaction products increases, and the system structure tends to be denser.

However, at the same M/P ratio, dry density decreased with extended curing age. By 14 d, the dry density of all mixes dropped to 481.1–499.7 kg/m^3^, a decrease of about 10–13%, and the gap between different M/P ratios narrowed significantly. This evolution pattern reflects the combined effect of two competing processes: on one hand, the continuous hydration reaction generates hydration products that fill pores, tending to increase density; on the other hand, water consumption and evaporation lead to increased porosity, reducing density. During long-term curing, the latter gradually becomes dominant. From a material design perspective, the effects of EP content and M/P ratio on dry density exhibit a “trade-off” pattern: EP content dominates the initial porosity of the system, while the M/P ratio regulates the amount of hydration products and the degree of densification. Their synergistic regulation is key to achieving a balance between coating lightness and mechanical properties.

### 3.4. Compressive Strength

**Figure 4:**
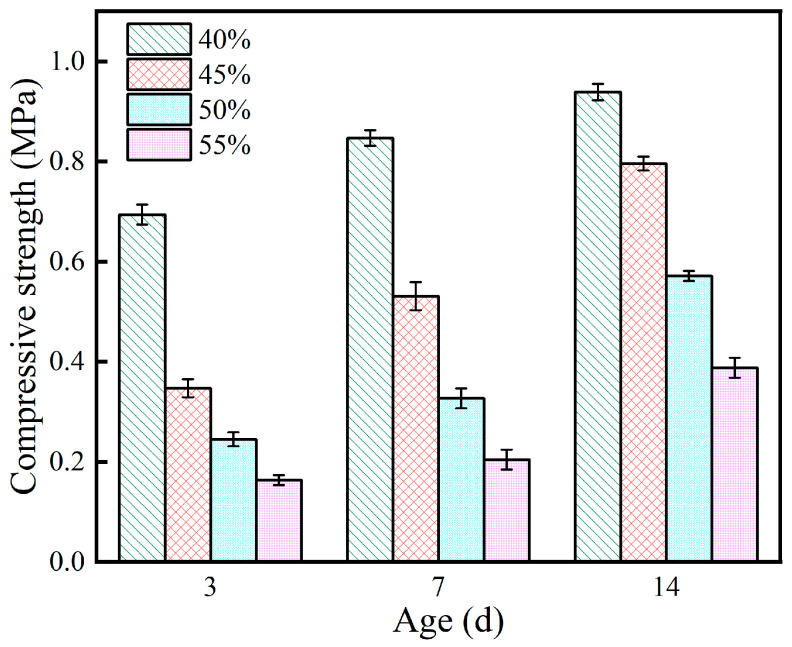
Effect of expanded perlite (EP) content on the compressive strength of MPC-based fireproof coatings at curing ages of 3, 7, and 14 days.

The effect of expanded perlite content on the compressive strength of the coating is shown in Figure 4. As EP content increased, the compressive strength of the specimens showed a continuous decreasing trend, and this pattern was consistent across all curing ages. Taking the 3 d age as an example, the compressive strength at 40% EP content was 0.69 MPa, while at 55% content, it plummeted to 0.16 MPa, a strength loss of up to 77%. By the 14 d age, the strength of the 40% content group increased to 0.94 MPa, while that of the 55% content group was only 0.39 MPa, with the absolute gap still significant.

The weakening effect of EP content on compressive strength mainly stems from two aspects: First, as a lightweight porous aggregate, EP itself has much lower strength than the MPC matrix, making it prone to become a weak point causing stress concentration under load. Second, the introduction of EP increases the system’s porosity, reducing the coating’s compactness and decreasing the effective load-bearing area. The combined effect of these two factors leads to a significant decrease in compressive strength with increasing EP content. This trade-off between lightweight properties and mechanical strength has been widely observed in EP-modified cementitious materials, where compressive strength decreases with increasing EP content due to the combined effects of aggregate weakness and increased porosity [8,14]. It is worth noting that although the strength of all content groups increased with extended curing age, the strength gap caused by EP content did not narrow. Taking the 40% and 55% content groups as examples, the strength difference at 3 d was 0.53 MPa, which expanded to 0.55 MPa at 14 d. This indicates that the negative impact of EP content on strength is persistent and difficult to compensate for by merely extending the curing age.

**Figure 5:**
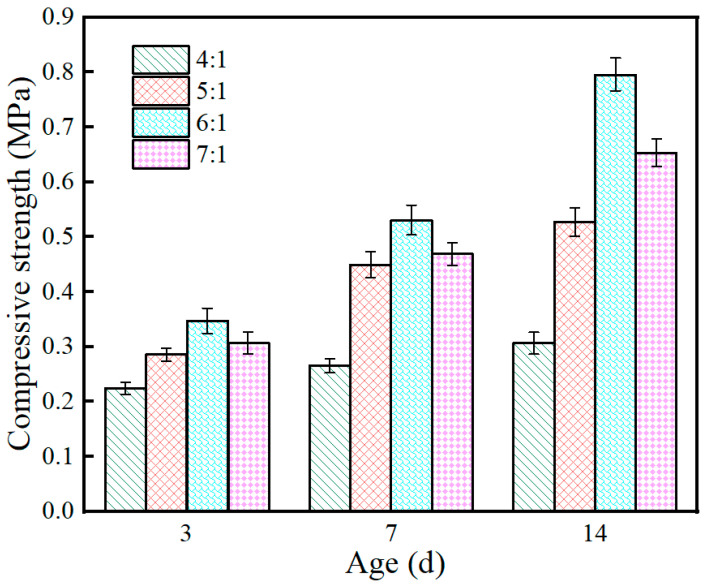
Effect of magnesium-to-phosphorus (M/P) ratio on the compressive strength of MPC-based fireproof coatings at curing ages of 3, 7, and 14 days.

The effect of the magnesium-to-phosphorus ratio on the compressive strength of the coating exhibited a non-monotonic “first increase then decrease” pattern, as shown in Figure 5. Taking the 14 d age as an example, the compressive strengths for M/P ratios of 4:1, 5:1, 6:1, and 7:1 were 0.31, 0.53, 0.80, and 0.65 MPa, respectively, with the 6:1 ratio peaking, an increase of about 160% compared to 4:1.

This pattern is closely related to the microstructural evolution of the MPC system. The compressive strength of the coating is mainly contributed by two types of components: the hydration product K-struvite provides cementing action, while unreacted magnesia particles act as a micro-skeleton. This dual-contribution mechanism has been documented in previous studies on MPC-based materials, where the balance between hydration products and residual MgO governs the mechanical performance [5,7,16]. When the M/P ratio is 4:1, phosphate is relatively abundant, leading to a higher amount of K-struvite formation. However, residual magnesia is insufficient to form a continuous skeleton support, resulting in relatively low strength. As the M/P ratio increases to 5:1, the proportion of K-struvite and residual magnesia tends to become more coordinated. They bond with each other to form a relatively dense microstructure, and the strength significantly improves. When the M/P ratio further increases to 6:1, the system reaches an optimal matching state: there is sufficient K-struvite to coat and bond aggregate particles, and an appropriate amount of residual magnesia to build a continuous skeleton, thus achieving the maximum compressive strength. This observation is supported by the XRD results (which will be discussed in Section 3.7.1), where the diffraction peaks of K-struvite are most pronounced at M/P = 6:1 compared to other ratios, indicating that the highest content of hydration product is formed under this condition, which directly contributes to the peak compressive strength. However, when the M/P ratio rises to 7:1, magnesia is excessive while hydration products are relatively insufficient. The excess unreacted particles cannot be effectively bonded, compromising matrix continuity, and strength decreases instead. It is also worth noting that the density difference between MgO (approximately 3.58 g/cm^3^) and KH_2_PO_4_ (approximately 2.34 g/cm^3^) may contribute to the observed strength trend. As the M/P ratio increases, the higher density MgO replaces the lower density KH_2_PO_4_ on a mass basis, resulting in a reduction in the total volume of the solid components. This volumetric change affects particle packing and the distribution of hydration products, potentially influencing the compactness and strength development of the matrix. The optimal balance at M/P = 6:1 may reflect not only the chemical balance between hydration products and residual MgO but also an optimal particle packing configuration. From a material design perspective, the compressive strength is optimal at an M/P ratio of 6:1, but considering bond strength and fire resistance comprehensively, the range of 5:1 to 6:1 also holds good potential for engineering application. This finding provides an important basis for the mix proportion optimization of the coating.

### 3.5. Bond Strength

**Figure 6:**
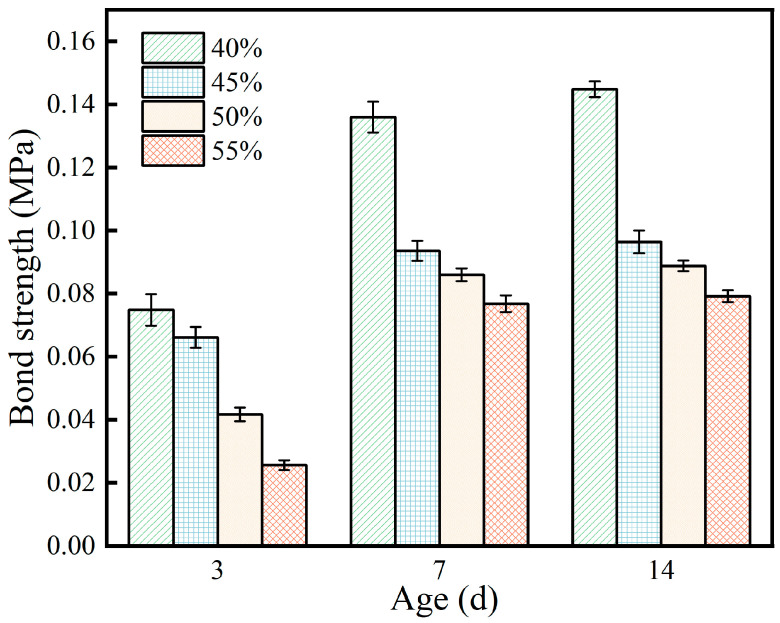
Effect of expanded perlite (EP) content on the bond strength between the coating and Q235 steel substrate at curing ages of 3, 7, and 14 days.

The effect of expanded perlite content on the bond strength between the coating and the steel plate substrate is shown in Figure 6. As EP content increased, bond strength showed a continuous decreasing trend, and this pattern was consistent across all curing ages. Taking the 14 d age as an example, the bond strength at 40% EP content was 0.14 MPa, while at 55% content, it decreased to 0.08 MPa, a strength loss of approximately 43%. Similar decreasing trends were observed at 3 d and 7 d ages.

The weakening effect of EP content on bond strength stems from the evolution of the microstructure in the interfacial zone. Bond strength mainly depends on two factors: the cohesive strength of the matrix itself and the physical and chemical bonding between the matrix and the steel plate. As EP content increases, the relative amount of MPC paste decreases, resulting in insufficient cementitious material capable of forming effective bonds with the steel plate, leading to a decrease in interfacial bonding force. This observation is consistent with previous findings that the bond strength of MPC-based coatings is highly dependent on the paste content and the quality of the interfacial transition zone [5,9]. Simultaneously, the enrichment of EP particles in the interfacial zone may form a weak transition zone, prone to interfacial debonding under stress.

**Figure 7:**
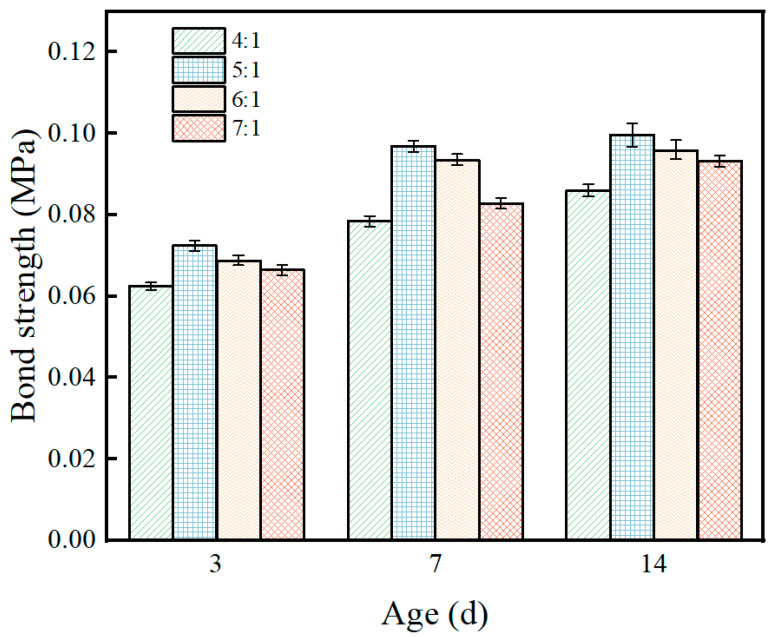
Effect of magnesium-to-phosphorus (M/P) ratio on the bond strength between the coating and Q235 steel substrate at curing ages of 3, 7, and 14 days.

It is worth noting that although the bond strength of all content groups improved with extended curing age, the strength gap caused by EP content persisted. Taking the 40% and 55% content groups as examples, the strength difference at 3 d was 0.04 MPa, which expanded to 0.06 MPa at 14 d. Based on the measurement uncertainty of ±0.005 MPa for bond strength and the standard deviations reported in Figure 7, these differences are statistically significant (*p* < 0.05). This indicates that the negative impact of EP on bond strength has a cumulative effect and is difficult to eliminate through prolonged curing.

The effect of the magnesium-to-phosphorus ratio on the bond strength of the coating exhibited a non-monotonic “first increase then decrease” pattern, as shown in Figure 7. Taking the 14 d age as an example, the bond strengths for M/P ratios of 4:1, 5:1, 6:1, and 7:1 were 0.082, 0.112, 0.105, and 0.095 MPa, respectively, with the 5:1 ratio peaking, an increase of about 37% compared to 4:1.

This pattern is closely related to the microstructural evolution of the interfacial transition zone. The formation of bond strength relies on two processes: the wetting and chemical bonding of the MPC matrix to the steel plate surface, and the encapsulation and cementation of EP particles by hydration products within the matrix. When the M/P ratio is 4:1, phosphate is relatively abundant, and the hydration reaction is faster, but the residual magnesia in the system is insufficient, leading to low cohesive strength of the matrix itself and limited interfacial bonding force. When the M/P ratio increases to 5:1, the system reaches an optimal balance: the amount of hydration product K-struvite formed is moderate, effectively wetting the steel plate surface and forming chemical anchorage; meanwhile, residual magnesia particles provide micro-skeleton support, making the interfacial transition zone dense and continuous. Therefore, the bond strength reaches its optimum. This result is consistent with SEM observations (which will be discussed in Section 3.7.2), where the interfacial zone of the 5:1 ratio sample appears uniform without obvious microcracks. When comparing the sample with M/P = 5:1 to that with M/P = 4:1, the microstructure at 5:1 exhibits a more uniform and denser morphology, suggesting a more controlled hydration process. The slower reaction kinetics at the optimal M/P ratio may allow for more selective formation of bonding species at the coating–steel interface, enhancing chemical anchorage and contributing to the peak bond strength observed in Figure 7. This interpretation is consistent with the notion that an appropriately balanced M/P ratio promotes the formation of a well-developed interfacial transition zone [5,16]. When the M/P ratio further increases to 6:1 and 7:1, bond strength decreases instead. This is because, with increasing magnesia excess, unreacted particles accumulate in the interfacial zone, disrupting matrix continuity; simultaneously, hydration products are relatively insufficient to fully encapsulate these particles, leading to a porous and loose interfacial transition zone and deteriorating bond performance.

From an engineering application perspective, bond strength is a key performance indicator for fireproof coatings, directly affecting the coating’s resistance to spalling under fire high temperatures. In this study, bond strength was optimal at an M/P ratio of 5:1, slightly different from the optimal compressive strength at 6:1. This “mismatch” phenomenon suggests that in actual mix proportion design, trade-offs need to be made based on performance requirements. Comprehensive consideration suggests that the range of 5:1 to 6:1 can serve as the preferred range for subsequent fire resistance studies.

### 3.6. Fire Resistance

**Figure 8:**
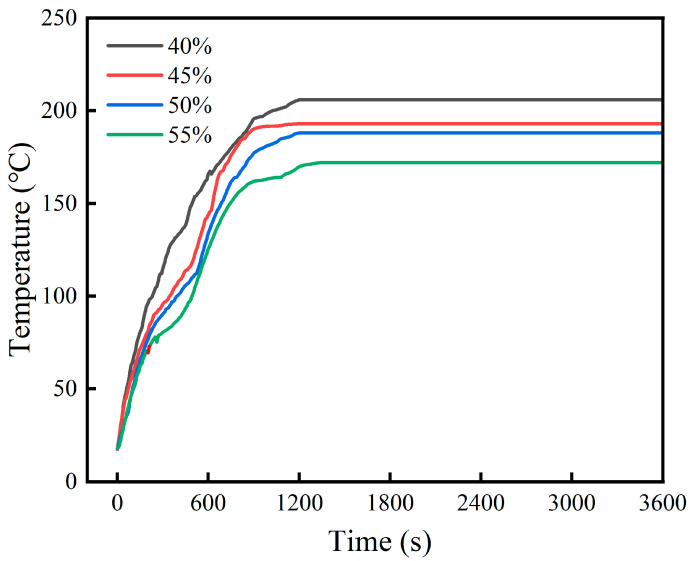
Effect of expanded perlite (EP) content on the back-side temperature of the steel plate during flame exposure.

The effect of expanded perlite content on fire resistance is shown in Figure 8. It can be seen from the figure that as the expanded perlite content increases, the back-side temperature of the steel plate gradually decreases. For expanded perlite contents of 40%, 45%, 50%, and 55%, after a combustion time of approximately 1200 s, the final stable back-side temperatures of the steel plates were 206 °C, 193 °C, 188 °C, and 172 °C, respectively. Moreover, the higher the expanded perlite content, the smaller the slope of the back-side temperature rise curve, i.e., the rate of temperature change per unit time decreases. This is because expanded perlite itself is a lightweight, porous material with low thermal conductivity, typically having a porosity between 70% and 90%, with internal pores filled with air. The air within the pores provides thermal insulation, making heat flow conduction difficult, and its thermal conductivity is much lower than that of ordinary cementitious materials. This thermal insulation mechanism has been well documented in studies on lightweight aggregates, where the high porosity and low thermal conductivity of EP contribute to enhanced fire resistance in cementitious composites [8,12,13]. When the content increases, the overall thermal conductivity of the material decreases, heat flow transfer is hindered, the back-side temperature rise slows down, and the curve slope decreases.

It is important to note that the back-side temperatures reported in Figure 8 represent the steady-state heat transfer condition under continuous flame exposure, not a true thermal equilibrium where the heat source is removed. Under steady-state heat transfer with a constant heat flux from the flame, the back-side temperature is governed by the total thermal resistance (R = L/k, where L is the coating thickness and k is the thermal conductivity) rather than by the thermal conductivity alone. Although higher EP content reduces the thermal conductivity k, it also significantly increases the overall thermal resistance due to the combined effects of lower k and the increased porosity of the coating. The coating with higher EP content (55%) exhibits a much higher thermal resistance than the coating with lower EP content (40%), resulting in a lower back-side temperature under sustained heating. This is analogous to a thermal insulation layer: a material with lower thermal conductivity does not necessarily “trap” heat; rather, it impedes heat flow, allowing less heat to reach the steel substrate. Therefore, the observed steady-state back-side temperatures (higher EP content → lower back-side temperature) are consistent with fundamental heat transfer principles. The transient behavior (slower temperature rise at higher EP content) and the steady-state behavior (lower final temperature at higher EP content) are both governed by the same underlying mechanism: the increased thermal resistance provided by the EP-rich coating.

**Figure 9:**
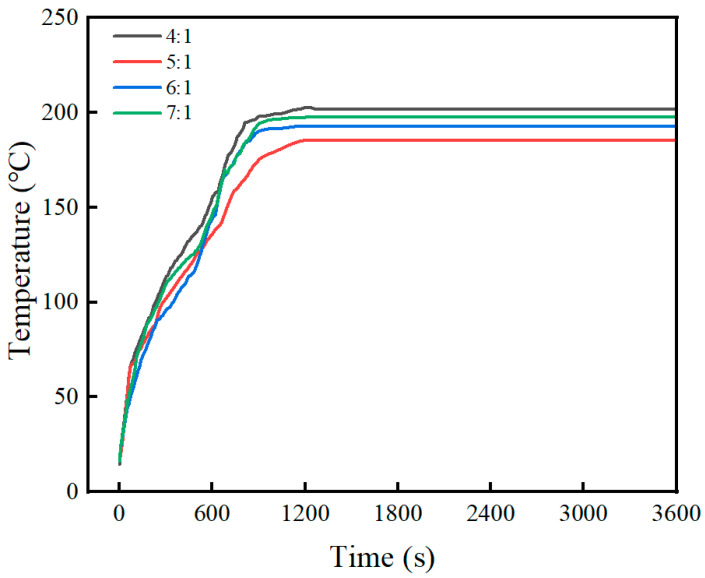
Effect of magnesium-to-phosphorus (M/P) ratio on the back-side temperature of the steel plate during flame exposure.

The effect of the magnesium-to-phosphorus ratio on fire resistance is shown in Figure 9. It can be seen from the figure that for M/P ratios of 4:1, 5:1, 6:1, and 7:1, the stable back-side temperatures of the steel plates at 1200 s were 202 °C, 180.4 °C, 193 °C, and 197.6 °C, respectively. Overall, the thermal insulation performance of the coating was closely related to the formation of the system’s hydration products and the matrix structure. When the M/P ratio was 4:1, although the amount of K-struvite formed was relatively high, residual magnesia was insufficient, and the skeleton effect was weak, resulting in an unstable overall coating structure, limited heat transfer resistance, and thus the highest back-side temperature. As the M/P ratio increased to 5:1, residual magnesia increased appropriately, forming a dense skeleton structure together with K-struvite, effectively improving the coating’s thermal barrier capacity, reducing the back-side temperature to the lowest, only 180.4 °C, demonstrating the best fire resistance. When the M/P ratio further increased to 6:1, the MgO content was too high. Although this could enhance the skeleton effect, the relative decrease in hydration products led to insufficient cementation, causing a decline in matrix compactness and an increase in back-side temperature to 193 °C. Under the 7:1 condition, this trend was more pronounced. Excess unreacted MgO could not form a continuous cementitious network, increasing heat transfer paths, and the final back-side temperature rose to 197.6 °C.

In addition to the steady-state back-side temperatures, the time required to reach critical temperature thresholds is an important parameter for fire protection evaluation according to international standards (e.g., ISO 834 [27], ASTM E119 [28]). From the temperature–time curves shown in Figure 8 and Figure 9, the optimal mix proportion (M/P = 5:1, EP = 45%) exhibited the longest time to reach elevated back-side temperatures, demonstrating superior thermal barrier performance. The slower temperature rise is attributed to the increased thermal resistance provided by the EP-rich coating and the favorable high-temperature phase evolution at this M/P ratio.

### 3.7. Hydration Products and Microstructure Analysis

#### 3.7.1. XRD Analysis

**Figure 10:**
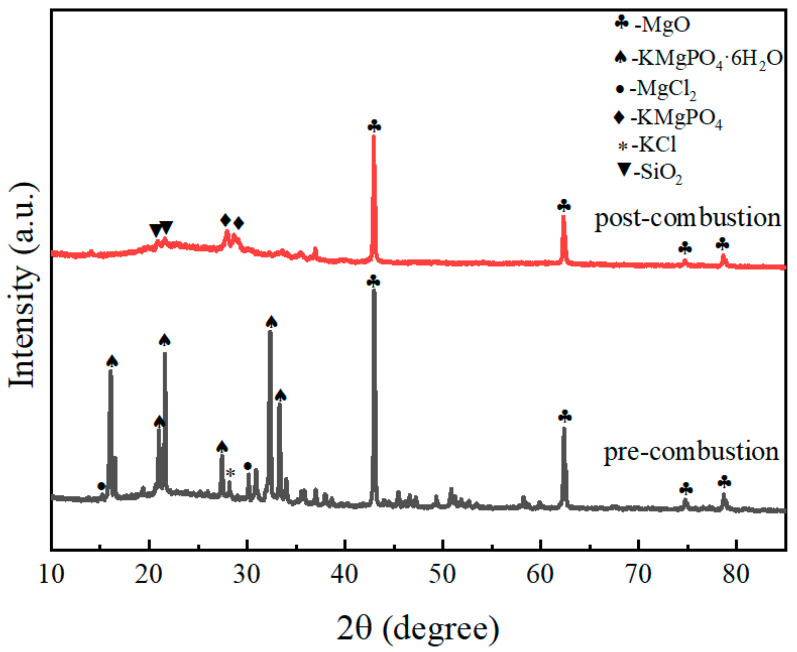
X-ray diffraction (XRD) patterns of MPC-based fireproof coating with 45% EP content and M/P = 5:1 at 7 days of hydration, before and after fire exposure.

The XRD patterns of the products of the magnesium phosphate cement-based coating with 45% expanded perlite content at a hydration age of 7 d, before and after combustion, are shown in Figure 10. It can be seen from the figure that in the XRD pattern before combustion, characteristic diffraction peaks of magnesium oxide (MgO), K-struvite (KMgPO_4_·6H_2_O), magnesium chloride (MgCl_2_), and potassium chloride (KCl) can be identified. Among these, K-struvite, as a typical hydrated phosphate phase, is the early hydration product of the system, endowing the coating with high early strength and compactness. MgCl_2_ and KCl mostly originate from residual salts during the preparation process. Although they help promote hydration at room temperature, their thermal stability is relatively poor. After combustion, the XRD pattern shows that the diffraction peaks of the hydrated phases completely disappear, replaced by anhydrous KMgPO_4_, indicating that K-struvite underwent dehydration and lattice rearrangement and transformed into the thermally stable anhydrous potassium magnesium phosphate phase. This phase transformation is consistent with previous studies on the thermal behavior of MPC, which reported that K-struvite decomposes to anhydrous KMgPO_4_ upon heating, contributing to the formation of a ceramic-like structure [7,8]. Simultaneously, the diffraction signals of MgCl_2_ and KCl were not significantly observed after combustion. It is speculated that they volatilized, melted, or underwent solid-state reactions with phosphates at high temperatures, becoming fixed, which is beneficial for improving system stability. Furthermore, the intensity of each characteristic peak decreased with flame combustion. At high temperatures, the amorphous SiO_2_ in expanded perlite may transform into crystalline SiO_2_, causing the diffraction peak of SiO_2_ in the XRD pattern to become more pronounced and sharper. Overall, the material’s phase evolution process at high temperatures achieved a transformation from “hydrated phases—volatile salts” to “anhydrous phosphates—heat-resistant oxides,” forming a thermally stable ceramized skeleton structure composed mainly of KMgPO_4_ and MgO.

**Figure 11:**
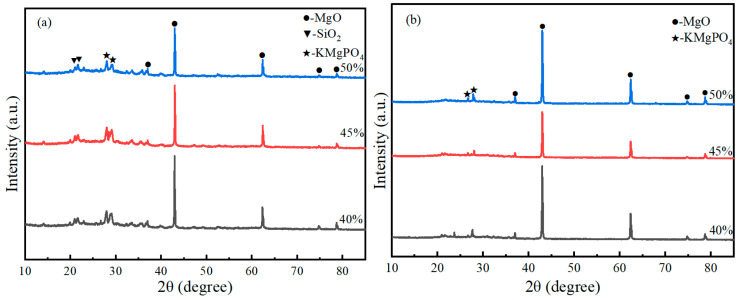
XRD patterns of the sample with variable expanded perlite content after combustion taken from the central heated zone: (**a**) flame-exposed surface layer; (**b**) steel-contact interface layer.

Figure 11 shows the XRD patterns of the expanded perlite-incorporated magnesium phosphate coating sample after the fire resistance test. All samples were taken from the central heated zone of the plate (the 50 mm × 50 mm area directly under the flame, as illustrated in Figure 1). Figure 11a corresponds to the flame-exposed surface layer (the side directly facing the flame), and Figure 11b corresponds to the steel-contact interface layer (the side adhered to the steel plate). The patterns display diffraction peaks of the main phases: MgO, KMgPO_4_, and a small amount of SiO_2_. In Figure 11a, at the flame-exposed end, the diffraction peaks are relatively pronounced, especially the peak for MgO at approximately 2θ = 42°. Magnesia is the main product of magnesium phosphate cement at high temperatures, and due to its good fire resistance, a relatively distinct diffraction peak is formed. Diffraction peaks for KMgPO_4_ appear in the 2θ = 25° to 30° range, indicating that the reaction between magnesium phosphate cement and potassium formed this phosphate phase in the flame-exposed zone. Diffraction peaks for SiO_2_ also begin to appear, suggesting that amorphous SiO_2_ from expanded perlite underwent transformation at high temperatures, forming crystalline SiO_2_, with clearly visible diffraction peaks. Figure 11b corresponds to the coating near the steel plate, where the intensity of the diffraction peaks is significantly weaker compared to that of the front end. Although the diffraction peak for MgO is still present, its intensity is relatively low, indicating that the temperature in this region was lower, and thus the formation of magnesia was more limited. Diffraction peaks for KMgPO_4_ are still visible in this region but with weakened intensity, indicating that some reaction also occurred at lower temperatures to form the potassium magnesium phosphate phase. Diffraction peaks for SiO_2_ still exist in the region near the steel plate, but due to the lower temperature in this area, silica mainly existed in an amorphous state, and no significant crystalline transformation occurred, resulting in weaker diffraction peaks.

**Figure 12:**
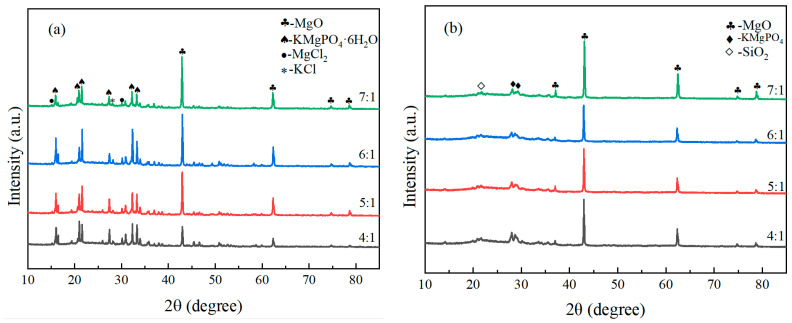
X-ray diffraction (XRD) patterns of MPC-based fireproof coatings with different M/P ratios (4:1, 5:1, 6:1, 7:1) and fixed EP content of 45%: (**a**) before combustion and (**b**) after combustion.

Figure 12 shows the XRD patterns of samples with different M/P ratios before and after fire exposure. It can be seen from the figure that there are significant differences in the phase composition of the coating before and after combustion under different M/P ratio conditions. Figure 12a is the XRD pattern of the coating samples before combustion, mainly composed of magnesium oxide (MgO), the hydration product K-struvite (KMgPO_4_·6H_2_O), and some unreacted by-products such as MgCl_2_ and KCl. As the M/P ratio increases, the diffraction peaks of MgO gradually strengthen, indicating that under higher-M/P-ratio conditions, the content of residual unreacted MgO is higher. The peak intensity of K-struvite is relatively more prominent at medium M/P ratios (e.g., 5:1, 6:1), indicating more sufficient reaction at these ratios, leading to the formation of more hydration products. Figure 12b is the XRD pattern of the coating samples after combustion. The diffraction peaks of K-struvite essentially disappear, transforming into thermally more stable KMgPO_4_ in the system, while retaining obvious diffraction peaks of MgO. This indicates that under high-temperature action, K-struvite undergoes dehydration and decomposition, generating anhydrous KMgPO_4_, thereby improving the high-temperature stability of the material. Diffraction peaks for SiO_2_ also begin to appear, forming crystalline SiO_2_ with clearly visible peaks. Comparing different mix ratios, it can be found that under the 6:1 condition, the peak intensities of KMgPO_4_ and residual MgO are relatively balanced, ensuring both skeleton stability and maintaining a moderate amount of reaction products, suggesting better structural compactness and fire stability. At 7:1, the MgO peak intensity is significantly enhanced, while KMgPO_4_ is relatively insufficient, which may lead to a decrease in structural continuity.

**Figure 13:**
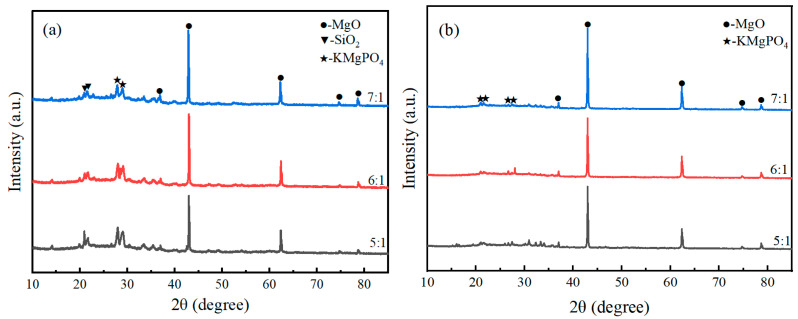
XRD patterns of the sample with variable M/P ratios after combustion taken from the central heated zone: (**a**) flame-exposed surface layer; (**b**) steel-contact interface layer.

Figure 13 shows the XRD patterns of the flame-exposed end (Figure 13a) and the steel plate contact end (Figure 13b) of samples with different M/P ratios after the fire resistance test. The patterns display diffraction peaks of the main phases: MgO, KMgPO_4_, and a small amount of SiO_2_. Figure 13a corresponds to the frontmost coating, which was directly exposed to the flame, subjected to higher temperatures and undergoing significant phase transformation. The diffraction peak for MgO is evident at 2θ ≈ 42°, indicating the formation of magnesia from the magnesium phosphate cement. Diffraction peaks for KMgPO_4_ appear in the 2θ ≈ 25–30° range, particularly at an M/P ratio of 6:1, where the peak for potassium magnesium phosphate is most significant, suggesting that more potassium magnesium phosphate formed at this ratio, contributing to stronger fire resistance. The peak for SiO_2_ can also be seen around 2θ ≈ 20°, indicating that SiO_2_ from expanded perlite formed crystalline SiO_2_, enhancing thermal insulation performance. In Figure 13b, for the coating near the steel plate, due to the lower temperature, the intensity of the MgO diffraction peak is weaker. Diffraction peaks for potassium magnesium phosphate are relatively clear at M/P ratios of 6:1 and 7:1, especially at 6:1, where more potassium magnesium phosphate formed, implying better fire resistance.

#### 3.7.2. SEM Analysis

**Figure 14:**
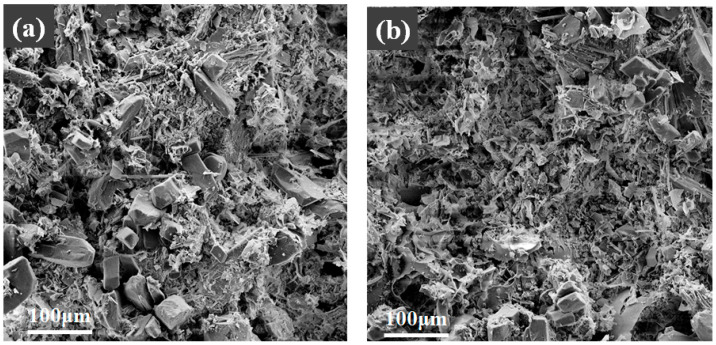
Scanning electron microscopy (SEM) images of the MPC-based fireproof coating with 45% EP content and M/P = 5:1: (**a**) before combustion and (**b**) after combustion.

The influence of 45% expanded perlite content on the fireproof coating before and after fire exposure is shown in Figure 14. Figure 14a displays an SEM image before combustion. It can be observed that the expanded perlite particles are uniformly distributed within the coating, and the surface morphology is relatively smooth, with no obvious cracks or voids between particles. This indicates that the unburned coating possesses good structural integrity, conducive to forming a uniform thermal protection layer. Additionally, the porous nature of expanded perlite may contribute to improving the coating’s thermal insulation properties. Figure 14b shows an SEM image after combustion. Compared to the image before combustion, the surface of the coating after combustion exhibits significant structural damage. The morphology of the expanded perlite particles has changed markedly, with many particles showing collapse or pulverization and cracks and pores appearing on the surface. These changes indicate that the coating underwent thermal decomposition and thermal stress effects under high-temperature conditions, leading to structural failure. Nevertheless, some expanded perlite particles still retain their basic morphology, possibly due to their low thermal conductivity and strong high-temperature resistance, enabling them to maintain their thermal barrier effect to a certain extent. In summary, the SEM images before and after combustion reflect the fire resistance of expanded perlite at high temperatures and its impact on the structural stability of the coating.

**Figure 15:**
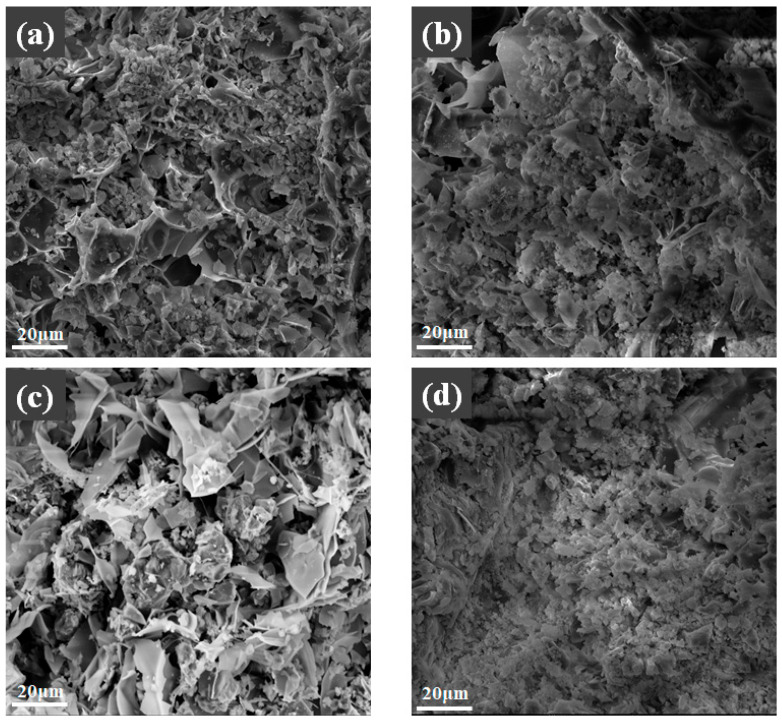
SEM images of fireproof coatings with different M/P ratios: (**a**) 4:1 before combustion, (**b**) 4:1 after combustion, (**c**) 5:1 before combustion, (**d**) 5:1 after combustion.

The effect of different magnesium-to-phosphorus ratios on the fire resistance of the fireproof coating with 45% expanded perlite content is shown in Figure 15. Figure 15a shows the sample before combustion with an M/P ratio of 4:1. The material’s structure appears relatively loose on the surface, with uneven particle distribution and numerous pores and voids, indicating its lower structural stability and poorer mechanical properties. In contrast, Figure 15c shows the sample before combustion with an M/P ratio of 5:1. The material’s structure is relatively uniform, with good bonding between particles. The surface is dense, and the structure is relatively complete, with no obvious cracks or voids between particles and fibers, indicating higher overall strength of the material. Comparing Figure 15c (M/P = 5:1) with Figure 15a (M/P = 4:1), the denser and more uniform microstructure at 5:1 suggests that the hydration process occurred at a more controlled rate at this ratio. The slower reaction kinetics may have allowed more time for the hydration products to selectively form and consolidate at the coating–steel interface, contributing to the superior bond strength observed at M/P = 5:1. This microstructural evidence supports the interpretation that an optimally balanced M/P ratio promotes the formation of a well-developed interfacial transition zone [5]. After combustion, the material with a 4:1 molar ratio (Figure 15b) exhibited significant damage, with extensive surface fragmentation and increased porosity, indicating poor stability at high temperatures and low mechanical strength. In contrast, the surface of the material with a 5:1 molar ratio (Figure 15d) still maintained good integrity. Although there were some small cracks and pores, the overall structure was stable, demonstrating strong fire resistance.

Comprehensive analysis shows that the material with a molar ratio of 5:1 maintained good structural integrity both before and after combustion, and its compressive strength was higher than that of the material with a molar ratio of 4:1. The uniform distribution and good bonding of the 5:1 molar ratio material endow it with stronger fire resistance and higher compressive strength, resulting in more excellent performance under high-temperature environments.

#### 3.7.3. Simultaneous Thermal Analysis (TGA)

**Figure 16:**
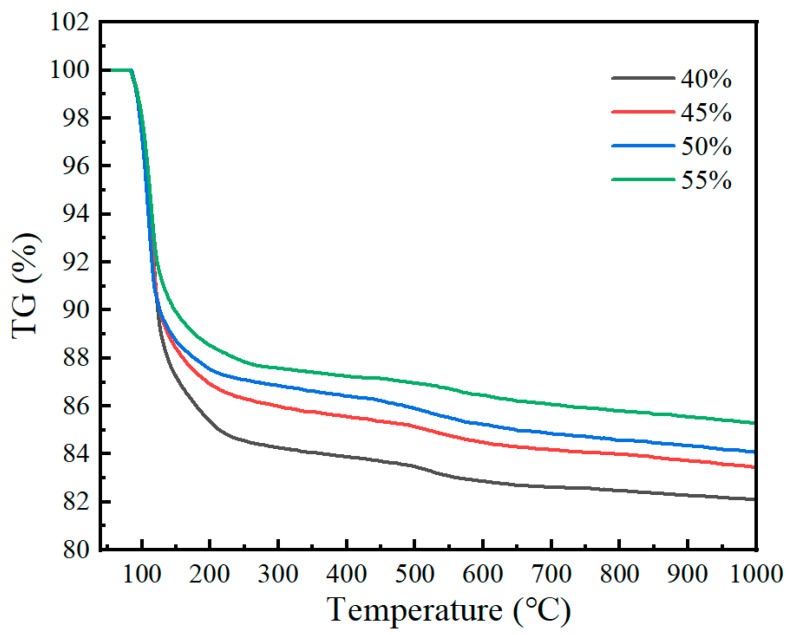
Thermogravimetric (TGA) curves of MPC-based fireproof coatings with different EP contents (40%, 45%, 50%, 55%) and fixed M/P = 5:1.

The TGA curves of coatings with different expanded perlite contents are shown in Figure 16. It can be seen from the figure that as the temperature increases, the mass of all samples decreases significantly. For expanded perlite contents of 40%, 45%, 50%, and 55%, the mass loss rates of the coatings are 17.62%, 16.23%, 15.59%, and 14.3%, respectively. Between 100 °C and 200 °C, an obvious mass loss phenomenon is observed. The mass loss at this stage may be related to water evaporation and the dehydration of the magnesium phosphate cement hydration product K-struvite. The decreasing slope of the curves becomes gentler with increasing expanded perlite content, indicating that when the expanded perlite content increases, the moisture content in the coating is relatively lower, and the rate of moisture volatilization is slower. As the temperature continues to rise, all curves tend to level off, indicating good thermal stability of the samples. The different expanded perlite contents have a relatively minor effect on the final mass loss of the samples. However, the higher the expanded perlite content is, the smaller the mass loss of the coating is. This may be due to the low thermal conductivity and good thermal insulation properties of expanded perlite, effectively reducing the impact of heat on the coating.

**Figure 17:**
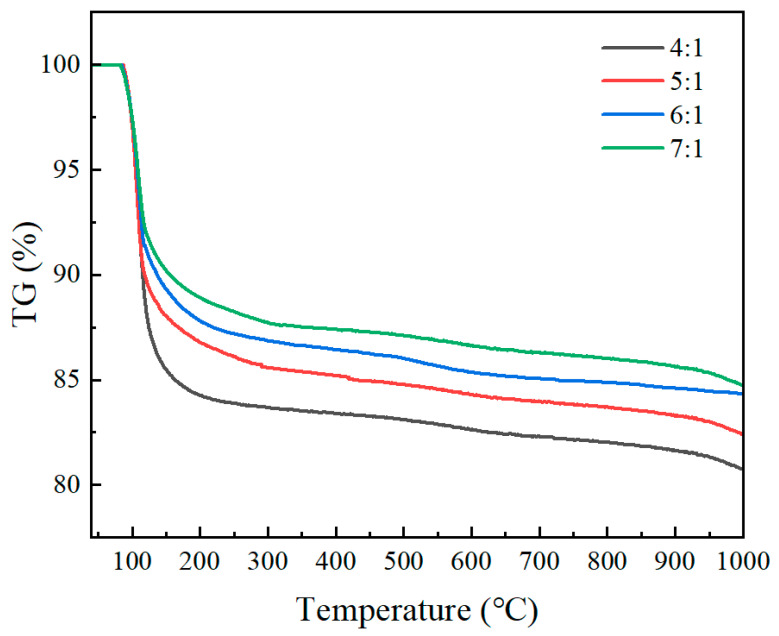
Thermogravimetric (TGA) curves of MPC-based fireproof coatings with different M/P ratios (4:1, 5:1, 6:1, 7:1) and fixed EP content of 45%.

The TG curves of coatings with different M/P ratios are shown in Figure 17. It can be seen from the figure that as the M/P ratio increases, the mass loss of the material during heating gradually decreases. At an M/P ratio of 4:1, the mass loss rate is 19.24%; at 5:1, it is 17.57%; at 6:1, it is 15.66%; and at 7:1, it is 15.24%. This trend indicates that samples with higher M/P ratios are more stable at high temperatures. In the curves for the 4:1 and 5:1 molar ratios, the mass loss is more significant, especially between 200 °C and 400 °C, indicating that materials with these ratios are more prone to thermal decomposition. In contrast, samples with 6:1 and 7:1 ratios show smaller mass loss, and the mass change tends to stabilize, especially after the temperature rises to 800 °C. The larger the M/P ratio, the smaller the mass loss upon heating, which suggests that the content of magnesium potassium phosphate hexahydrate decreases. Analysis reveals that under the four tested M/P ratios, the amount of magnesium oxide is excessive compared to the amount of phosphate. Therefore, the amount of K-struvite formed is closely related to the phosphate content. As the M/P ratio increases, the amount of phosphate gradually decreases, leading to a gradual decrease in the amount of K-struvite formed.

## 4. Discussion

### 4.1. Optimal Expanded Perlite Content

The experimental results demonstrate that EP content plays a dominant role in balancing the thermal insulation and mechanical properties of MPC-based fireproof coatings. As EP content increased from 40% to 55%, dry density decreased by 21.2% and back-side temperature dropped by 34 °C, while compressive strength decreased by 77%. This trade-off between insulation efficiency and mechanical performance is consistent with previous studies on lightweight aggregates in cementitious composites. Fang et al. [8] reported that incorporating EP into MPC decreased the fluidity, extended the setting time, and enhanced the adhesive strength before and after fire testing. Under fire exposure, the MKPC-EP composite exhibited a much denser microstructure compared to the MKPC paste, with interconnecting MgKPO_4_·H_2_O of radiative flakes [8]. Similarly, Fang et al. [14] demonstrated that surface modification of EP using silane impregnation solution, water glass, or stearic acid can further improve the performance of MPC-based fireproof mortars, with silane impregnation showing the greatest effectiveness in enhancing compressive strength and fire resistance [14].

The optimal EP content identified in this study (45%) differs from some previous reports. For instance, Qian et al. [18] found that an EP content of 40% yielded optimal overall performance in MPC coatings utilizing waste magnesia–carbon bricks. This discrepancy may be attributed to differences in the characteristics of the EP used (particle size distribution, bulk density, and water absorption) and the specific MPC matrix composition. In the present study, the higher optimal EP content (45%) is likely due to the use of dead burnt magnesia with lower reactivity, which allowed for better workability and interfacial bonding at higher aggregate contents. Furthermore, the “internal curing” effect of EP—where absorbed water is gradually released during hydration—may have contributed to improved matrix densification at moderate EP levels, as suggested by the water demand analysis in Section 3.1.

### 4.2. Influence of Magnesium-to-Phosphorus Ratio

The M/P ratio exhibited a non-monotonic influence on both compressive strength and bond strength, with compressive strength peaking at M/P = 6:1 (0.80 MPa) and bond strength peaking at M/P = 5:1 (0.097 MPa). This “mismatch” between the optimal ratios for different properties reflects the competing roles of hydration products and residual MgO in the matrix structure. At lower M/P ratios (4:1), the abundance of phosphate promotes rapid formation of K-struvite, but insufficient residual MgO limits the development of a continuous micro-skeleton. As the M/P ratio increases to 5:1–6:1, the balance between K-struvite (providing cementing action) and residual MgO (acting as micro-skeleton) reaches an optimal state, resulting in peak mechanical performance. At higher M/P ratios (7:1), excessive MgO leads to poor matrix continuity and reduced strength.

These findings are consistent with recent studies on M/P ratio effects in MPC systems. Previous research has demonstrated that the M/P ratio significantly influences the bond strength and compressive strength of MPC-based fireproof coatings, with optimal performance typically achieved within the range of 5:1 to 6:1 [16,18]. Hu et al. [16] reported that the M/P ratio plays a critical role in determining the bond strength of MPC-based steel fireproof coatings. Similarly, Li et al. [18] observed that the mechanical properties of MPC coatings are closely related to the M/P ratio, with different optimal ratios for compressive strength and bond strength. The correlation between the XRD results (Figure 12a) and compressive strength (Figure 5) further supports this interpretation: the maximum K-struvite diffraction intensity observed at M/P = 6:1 coincides with the peak compressive strength, confirming that the hydration product content is a key determinant of compressive performance. Conversely, the bond strength optimum at M/P = 5:1, while not corresponding to the maximum hydration product content, reflects the importance of a well-developed interfacial transition zone. This pattern can be explained by the differential sensitivity of bond strength to interfacial properties: bond strength relies more heavily on the wetting and chemical anchorage at the steel interface, which is optimized when hydration products are sufficiently abundant to form a dense interfacial transition zone (M/P = 5:1). In contrast, compressive strength benefits more from the micro-skeleton provided by residual MgO particles, which becomes more pronounced at slightly higher M/P ratios (6:1).

### 4.3. High-Temperature Phase Evolution and Ceramic Skeleton Formation

The XRD and TGA analyses revealed a distinct phase evolution pathway in the EP-MPC composite system under high-temperature exposure. K-struvite undergoes dehydration between 200 and 400 °C, transforming into anhydrous KMgPO_4_, while residual MgO remains stable throughout the heating process. This transformation pattern is consistent with previous studies on pure MPC systems. Fang et al. [8] reported that during elevated temperature exposure, MgKPO_4_·6H_2_O (K-struvite) decomposes by losing crystal water stage by stage, transforming into MgKPO_4_·H_2_O. Gardner et al. [7] reported that K-struvite decomposition in MPC binders occurs in the range of 150–300 °C, with the formation of amorphous and crystalline phases depending on the heating rate and final temperature. Yu et al. [20] investigated the behavior of MPC with sulphoaluminate cement at elevated temperatures and observed thermal decomposition of hydration products within a similar temperature range. The present study confirms that this transformation is preserved in EP-MPC composites, with the additional contribution of EP-derived SiO_2_.

A notable finding of this study is the crystallization of amorphous SiO_2_ from EP at temperatures above 400 °C, as evidenced by the appearance of distinct SiO_2_ diffraction peaks in the post-combustion XRD patterns (Figure 10, Figure 11, Figure 12 and Figure 13). This phenomenon has been reported in other cementitious systems incorporating EP. For instance, Demirboa R et al. [23] observed that the incorporation of expanded perlite influenced the thermal conductivity and mechanical properties of geopolymer composites, with the amorphous SiO_2_ in EP undergoing structural changes at elevated temperatures. Fang et al. [8] observed the formation of a denser microstructure in MKPC-EP composites after exposure to flame, with interconnecting MgKPO_4_·H_2_O of radiative flakes. The present study provides direct evidence that EP-derived SiO_2_ undergoes a phase transition from amorphous to crystalline form at elevated temperatures, contributing to the densification of the ceramic skeleton. This additional densification mechanism may explain why the EP-MPC composite maintained structural integrity even after prolonged flame exposure, with back-side temperatures well below the critical threshold for steel strength degradation (approximately 300 °C).

### 4.4. Fire Resistance Performance in Context

The best fire resistance performance in this study was achieved at M/P = 5:1 with 45% EP content, yielding a back-side temperature of 180.4 °C after 1200 s of flame exposure. This performance compares favorably with values reported in the literature. Previous studies on EP-MPC composites have demonstrated the potential of such materials for fire protection applications, with the addition of EP contributing to enhanced thermal insulation and improved structural stability under fire exposure [8,14]. Li et al. [19] investigated brucite-based MPC fire-resistive coatings and reported favorable fire resistance performance. Li et al. [18] developed MPC fire-resistive coatings utilizing waste magnesia–carbon bricks and demonstrated their effectiveness for steel structure protection.

The superior fire resistance observed in the present study (180.4 °C) can be attributed to the synergistic optimization of both EP content and M/P ratio. The combination of 45% EP (providing optimal thermal insulation) and M/P = 5:1 (yielding the most favorable high-temperature phase evolution and ceramic skeleton densification) resulted in a composite coating with exceptional thermal barrier performance. From a heat transfer perspective, the coating acts as a thermal barrier that impedes heat flow from the flame to the steel substrate. The thermal resistance increases with EP content due to the combined effects of lower intrinsic thermal conductivity of EP particles and the increased porosity of the coating. Under steady-state-heating conditions, the back-side temperature is determined by the total thermal resistance across the coating thickness; a higher thermal resistance results in a lower back-side temperature, consistent with the trend observed in Figure 8. The relatively low back-side temperature indicates that the coating effectively maintained the steel substrate below 200 °C, well within the safe operating range for steel structures (below 300 °C). This performance meets or exceeds the requirements for standard fire protection applications.

### 4.5. Implications for Mix Proportion Design

The comprehensive evaluation of EP content and M/P ratio in this study provides a systematic basis for the mix proportion design of MPC-based fireproof coatings. The findings suggest that a two-step optimization approach is most effective: first, select EP content based on the required balance between thermal insulation and mechanical properties (with 45% as the recommended baseline); second, adjust M/P ratio according to the priority of performance requirements (M/P = 5:1 for bond strength and fire resistance, or M/P = 6:1 for compressive strength). This design framework enables targeted optimization for specific application scenarios.

From a practical engineering perspective, the optimal mix proportion (M/P = 5:1, EP = 45%) exhibits several favorable characteristics for real-world application. First, the fluidity of approximately 260 mm (Section 3.2) is suitable for spray application, which is the predominant construction method for fireproof coatings on steel structures. Second, the dry density of approximately 560 kg/m^3^ falls within the range of lightweight fireproof coatings, facilitating ease of handling and reducing structural dead load. Third, the bond strength of 0.097 MPa and back-side temperature of 180.4 °C under fire exposure demonstrate that the coating can effectively protect steel substrates under standard fire conditions. Fourth, all raw materials (dead burnt magnesia, potassium dihydrogen phosphate, expanded perlite) are commercially available and cost-effective, supporting the potential for large-scale production.

Furthermore, the insights gained from microstructural analysis—particularly the role of EP-derived SiO_2_ crystallization in enhancing high-temperature stability—suggest that future research could explore the use of pre-treated or surface-modified EP to further enhance the ceramic skeleton formation. The modification approach reported by Fang et al. [14], using silane impregnation solution, water glass, or stearic acid for surface hydrophobicity modification of EP particles, represents a promising direction for further performance enhancement. Among these, silane impregnation was found to be most effective in improving the compressive strength and fire resistance of MPC-based fireproof mortars [14].

### 4.6. Environmental Considerations

The performance of MPC-based materials is known to be sensitive to environmental conditions, particularly temperature and humidity, which influence both the hydration kinetics during curing and the long-term durability of the coating. In this study, all experimental procedures were conducted under controlled laboratory conditions (23 ± 2 °C, 60 ± 5% RH) to minimize environmental variability. However, it is important to discuss how these factors may affect the results and their implications for practical applications.

#### 4.6.1. Influence of Ambient Temperature

The hydration reaction of MPC is exothermic and proceeds rapidly at elevated temperatures. Under higher ambient temperatures (e.g., >30 °C), the reaction rate accelerates, leading to faster setting and potentially reduced workability. Conversely, at lower temperatures (<10 °C), the reaction slows considerably, which may delay strength development and prolong the curing period. The controlled temperature (23 ± 2 °C) used in this study represents typical indoor conditions and allows for reproducible hydration kinetics. For field applications, it is recommended to apply the coating within a moderate temperature range (10–30 °C) to ensure consistent performance. In colder climates, the use of warm mixing water or the addition of accelerators may be necessary to maintain adequate reaction rates.

#### 4.6.2. Influence of Ambient Humidity

Relative humidity affects both the water demand during mixing and the drying behavior of the coating after application. As discussed in Section 3.1, the water absorption capacity of EP is influenced by ambient humidity; under dry conditions, EP particles exhibit higher suction, increasing the initial water demand. During curing, low ambient humidity accelerates moisture evaporation, which may lead to increased shrinkage and microcracking, particularly in coatings with high EP content. Conversely, high humidity may prolong the curing period and delay the development of mechanical properties. In this study, the controlled relative humidity (60 ± 5%) provided a balanced environment that allowed for consistent hydration and drying without excessive shrinkage or delayed strength gain. For on-site application, it is advisable to protect the applied coating from direct sunlight and strong wind during the initial curing period to prevent rapid moisture loss.

#### 4.6.3. Implications for Practical Application

The environmental dependencies identified in this study have important implications for the practical application of the developed EP-MPC fireproof coating:

Seasonal variations: Application during summer (high temperature) may require the use of retarders to extend working time, while winter application (low temperature) may necessitate preheating of mixing water or the use of accelerators to maintain adequate reaction rates.

Humidity control: In arid regions, additional measures such as covering the coated surface with plastic sheeting during the first 24–48 h may be necessary to prevent rapid moisture loss and ensure proper hydration.

Quality assurance: Monitoring of ambient conditions (temperature and humidity) during application should be considered as part of quality control protocols to ensure consistent coating performance.

These considerations are particularly relevant for the practical deployment of the developed coating, and the experimental results presented in this study—obtained under controlled conditions—provide a reliable baseline for performance expectations under typical application environments.

## 5. Limitations of the Study

While this study provides valuable insights into the composition optimization and performance mechanisms of EP-reinforced MPC fireproof coatings, several limitations should be acknowledged. The experimental design assumes homogeneous material properties and uniform hydration under controlled laboratory conditions (23 ± 2 °C, 60 ± 5% RH), which may not fully replicate the variable temperature, humidity, and curing conditions encountered in field applications. All tests were conducted on laboratory-scale specimens, and the fire resistance assessment employed a butane flame that simulates standard fire scenarios but does not account for complex real-fire conditions such as varying heating rates or mechanical loading. The raw materials used (dead burnt magnesia from a single source, a specific type of expanded perlite) and the investigated ranges of M/P ratio (4:1–7:1) and EP content (40–55%) may restrict the direct generalization of the results to other material sources or mix proportions. Additionally, this study focused on early-age performance (up to 14 days) without evaluating long-term durability aspects such as water resistance, freeze–thaw cycles, or weathering. Acknowledging these limitations does not diminish the validity of the findings but rather provides a transparent basis for interpreting the results and guiding future research toward full-scale validation, long-term durability assessment, and field application trials.

## 6. Conclusions

This paper systematically studied the effects of expanded perlite content and magnesium-to-phosphorus ratio on the performance of magnesium phosphate cement-based fireproof coatings for steel structures. Combined with microstructural analysis, the mechanism of performance evolution was revealed. The main conclusions are as follows:

(1) EP content dominates the balance between the coating’s thermal insulation performance and mechanical properties by regulating the pore structure. Increasing EP content from 40% to 55% reduced the coating’s dry density by 21% and lowered the back-side temperature by 34 °C but decreased compressive strength by 77%. Considering overall performance, 45% is the optimal EP content, achieving the best thermal insulation efficiency while ensuring basic mechanical properties.

(2) The M/P ratio determines the coating’s interfacial bond strength and high-temperature stability by regulating the proportion of hydration products and residual MgO. As the M/P ratio increased from 4:1 to 7:1, both compressive strength and bond strength showed a “first increase then decrease” trend. Bond strength was optimal at 5:1 (0.097 MPa), while compressive strength peaked at 6:1 (0.80 MPa). The 5:1 ratio exhibited the densest ceramized structure and the lowest back-side temperature (180.4 °C) at high temperatures, indicating that a system with a balanced reaction is more conducive to forming a stable high-temperature skeleton.

(3) The MPC-EP composite system undergoes a phase evolution path of “hydrated phase dehydration → residual salt volatilization → ceramic skeleton formation” at high temperatures. K-struvite dehydrates and transforms into anhydrous KMgPO_4_ between 200 and 400 °C, forming a ceramic skeleton together with residual MgO. The amorphous SiO_2_ in EP crystallizes above 400 °C, further densifying the structure. This evolution process is the fundamental reason for the coating maintaining high-temperature structural stability.

(4) Based on a comprehensive performance evaluation, the basic mix proportion for the MPC-based fireproof coating for steel structures is determined as: an M/P ratio of 5:1, magnesium phosphate cement content of 55%, and expanded perlite content of 45%. Under this mix proportion, the coating has a dry density of approximately 560 kg/m^3^, a 14 d compressive strength of 0.53 MPa, a bond strength of 0.097 MPa, and a back-side temperature of 180.4 °C in the fire resistance test, meeting the basic requirements for fire protection of steel structures.

## Figures and Tables

**Table 1:** Chemical composition of magnesia/%.

Oxides	MgO	SiO_2_	CaO	Fe_2_O_3_	Other
Content/%	97.1	0.9	1.5	0.3	0.2

**Table 2:** Chemical composition of potassium dihydrogen phosphate/%.

KH_2_PO_4_	Water Insoluble	Water Content	pH
≥99.5	≤0.1	≤0.1	4.2–4.5

**Table 3:** Expanded perlite parameters.

Particle Size Distribution (Passing 1–3 mm Sieve, %)	Thermal Conductivity W/(m·K)	Bulk Density (kg/m^3^)	Moisture Content (%)
70–90	0.04	96	0.27

**Table 4:** Chemical composition of expanded perlite/%.

Oxides	SiO_2_	Al_2_O_3_	Fe_2_O_3_	CaO	K_2_O	Na_2_O	MgO	TiO_2_
Content/%	73.2	13.7	0.98	0.82	3.12	2.53	0.18	0.09

**Table 5:** Mix proportion design.

No.	M/P Ratio	Expanded Perlite Content/%	Magnesium Phosphate Cement Content/%	Borax (wt.% of MgO)
1	6	40%	60%	10%
2	6	45%	55%	
3	6	50%	50%	
4	6	55%	45%	
5	4	45%	55%	
6	5	45%	55%	
7	7	45%	55%	

Note: Borax was added at a fixed dosage of 10% by weight of MgO for all mix proportions, calculated separately from the percentages shown in the table.

**Table 6:** Effect of expanded perlite content on water consumption at fixed fluidity.

Expanded Perlite Content	40%	45%	50%	55%
Fluidity (mm)	255 ± 3	259 ± 4	258 ± 3	260 ± 3
Water Consumption (g)	750 ± 8	850 ± 9	970 ± 11	1070 ± 12

Note: Values are presented as mean ± standard deviation (*n* = 3).

**Table 7:** Effect of M/P ratio on fluidity.

M/P Ratio	4:1	5:1	6:1	7:1
Fluidity (mm)	270	262	259	251

## Data Availability

The original contributions presented in this study are included in the article. Further inquiries can be directed to the corresponding author.
